# Use of Subperiosteal Drain Versus Subdural Drain in Chronic Subdural Hematomas Treated With Burr-Hole Trepanation: Study Protocol for a Randomized Controlled Trial

**DOI:** 10.2196/resprot.5339

**Published:** 2016-04-08

**Authors:** Jehuda Soleman, Katharina Lutz, Sabine Schaedelin, Luigi Mariani, Javier Fandino

**Affiliations:** ^1^Department of Neurosurgery, Kantonsspital Aarau, Aarau, SwitzerlandAarauSwitzerland; ^2^Department of Neurosurgery, University Hospital Basel, Basel, SwitzerlandBaselSwitzerland; ^3^Clinical Trial Unit, University Hospital of Basel, Basel, SwitzerlandBaselSwitzerland

**Keywords:** chronic subdural hematoma, drain, hematoma, recurrent hematoma, burr-hole trepanation

## Abstract

**Background:**

Chronic subdural hematoma (cSDH) is one of the most frequent neurosurgical conditions affecting elderly people and is associated with substantial morbidity and mortality. The use of a subdural drain (SDD) after burr-hole trepanation for cSDH was proven to reduce recurrence and mortality at 6 months. To date in neurosurgery practice, evidence-based guidelines on whether an SDD or subperiosteal drain (SPD) should be used do not exist. Currently both methods are being practiced depending on the institute and/or the practicing neurosurgeon.

**Objective:**

The aim of this study is to compare the reoperation rates after burr-hole trepanation and insertion of an SPD or SDD in patients with cSDH.

**Methods:**

This is a prospective, noninferiority, multicenter, randomized controlled trial designed to include 220 patients over the age of 18 years presenting with a symptomatic cSDH verified on cranial computed tomography or magnetic resonance imaging who are to undergo surgical evacuation with burr-hole trepanation. After informed consent is obtained, patients are randomly allocated to an SPD or SDD group. The primary endpoint is recurrence indicating a reoperation within 12 months.

**Results:**

This research is investigator-initiated and has received ethics approval. Patient recruitment started in April 2013, and we expect all study-related activities to be completed by the end of 2016 or beginning of 2017.

**Conclusions:**

To date, evidence-based recommendations concerning the operative treatment of cSDH are sparse. Results of this research are expected to have applications in evidence-based practice for the increasing number of patients suffering from cSDH and possibly lead to more efficient treatment of this disease with fewer postoperative complications.

**Trial Registration:**

ClinicalTrials.gov NCT01869855; https://clinicaltrials.gov/ct2/show/NCT01869855 (Archived by WebCite at http://www.webcitation.org/6fNK4Jlxk)

## Introduction

### Background

Chronic subdural hematoma (cSDH) is one of the most frequent neurosurgical conditions affecting elderly people and is associated with substantial morbidity and mortality [1-3]. Its incidence is reported to be 1.7-13.1 per 100,000 inhabitants per year, but the incidence has been steadily increasing due to prolonged life expectancy [4-6]. Surgical treatment is recommended in patients with neurological symptoms. In the only evidence-based review of the different surgical treatment modalities of cSDH, Weigel et al concluded that burr-hole craniostomy with irrigation and drainage has the best cure-to-complication ratio [7]. Recurrence is the most common complication following surgical treatment of cSDH with a rate of 0%-30% [3,4,8]. A randomized controlled study by Santarius et al showed reduced recurrence and mortality placing a subdural drain (SDD) compared to not placing a drain after burr-hole evacuation of cSDH [2]. Gazzeri et al and Zumofen et al used a closed subperiosteal drain (SPD) instead of the more commonly used SDD, and the method showed equal or superior results in outcome, complications, and postoperative symptoms compared to previous studies [9,10]. Since the SPD is not positioned in direct contact to cortical structures, bridging veins, or hematoma membranes, it is considered safer and might be favorable to an SDD. In a retrospective study, Bellut et al compared 48 patients treated with SPD to 65 patients treated with SDD and found lower mortality rates and fewer serious complications in the group treated with SPD with no difference in recurrence rate of cSDH [5]. However, none of the results showed a significant difference, and it was concluded that further randomized studies with a larger patient cohort are needed [5]. In a recently published prospective randomized study, Kaliaperumal et al concluded that the recurrence rate after placing an SPD is equal to that following placement of an SDD, with the modified Rankin scale (mRS) of the patients in the SPD group being significantly better after 6 months [11]. However, the results may have been biased since the preoperative mRS in the SPD group was inferior to those in the SDD group. In addition, the number of patients studied was small (25 per group), and the overall recurrence rate was 0%, with very low morbidity and mortality rates compared to the literature. Due to these biases the authors recommend further prospective and randomized studies with larger patient cohorts [11].

At the time of this writing, evidence-based guidelines on which method should be used in cSDH do not exist, and both SDDs and SPDs are being used depending on the institute and/or the practicing neurosurgeon.

### Aims and Objectives

The primary objective of our study is to investigate in a randomized controlled fashion whether the recurrence rate after insertion of an SPD is noninferior compared to the insertion of an SDD in patients undergoing surgical evacuation of a cSDH with burr-hole trepanation. The secondary objective of the study is to assess whether the insertion of an SPD leads to fewer operative complications, a lower mortality, and a better outcome.

## Methods

### Trial Design

This is a prospective, multicenter, noninferiority, randomized controlled study. Eligible participants are block-randomized in a 1:1 allocation ratio to one of two arms: an intervention arm, insertion of an SPD and a control arm, insertion of an SDD.

### Study Setting and Selection Criteria

Patients will be recruited from the departments of neurosurgery at Kantonsspital Aarau and University Hospital of Basel in Switzerland. Both centers are major trauma and neurosurgical referral centers. Eligible participants are female or male over the age of 18 years presenting to one of the centers with a symptomatic cSDH diagnosed by computed tomography (CT) and/or magnetic resonance imaging (MRI). Exclusion criteria are as follows: (1) surgeon decides to perform a craniotomy based on any intraoperative condition (eg, acute hematoma), (2) cSDH is caused by an underlying condition (eg, overdrainage of a ventriculoperitoneal shunt), and (3) no informed consent.

### Informed Consent

Written informed consent of the patient or relative must be obtained by a member of the neurosurgical staff prior to randomization. The neurosurgical staff members undergo a trial-specific training making them eligible to include patients in the trial. A written information sheet is given to the patient or relative and as much time as necessary is allowed to discuss the options. If the patient is unable to give consent due to the nature of the hemorrhage, a personal representative is approached to give consent on behalf of the patient. If the patient is unable to consent and a relative or representative of the patient is not available, an independent doctor can consent on behalf of the patient. In such a case, consent by the patient or representative must be sought at a later time or the patient will be excluded from the study. The consent forms (written in German) are filed with the trial documentation.

### Randomization

Randomization with blocks of 30 in an allocation ratio of 1:1 will be performed by the investigators using the Web-based randomization software Random Allocation version 1.0. Instructions on which drain should be implanted are kept in sealed envelopes labeled with sequential study numbers and opened at surgery before the insertion of the drain. The nature of this intervention does not allow for masking of treatment allocation. However, data is encoded and clinicians are masked to outcomes when possible.

### Trial Interventions

All patients undergo surgical evacuation of a symptomatic cSDH with two burr-hole trepanations; an SPD or SDD is then inserted without suction according to the arm of the study to which the patient has been randomized. The surgical procedure is standardized for both institutions and consists of supine positioning of the patients on a horseshoe headrest. The frontal and parietal areas of the head are shaved, and patient is draped. After skin incision, two 13 mm burr-holes about 7 to 8 cm apart are drilled over the maximum width of the hematoma. The dura mater is opened with a cruciate incision and coagulated. The subdural hematoma is then washed out with warm saline with or without a Nelathon catheter. Once the surgeon completes the irrigation and is ready for drain insertion, the randomization envelope is opened and the assigned drain (subdural or subperiosteal) is inserted. The SDD is inserted from the parietal burr-hole in frontal direction under visual control. The SPD is inserted subgalealy and placed over both burr-holes. In case of a subperiosteal insertion, the burr-holes should not be sealed off with any kind of material (eg, PDS-Folie, Spongostan) so that a communication between the subdural space and the SPD is maintained. Bilateral hematomas are treated as one case; both sides receive the same treatment. Patients with crossover treatment (ie, a patient is randomized to SDD but the surgeon feels it is unsafe to insert an SDD because the brain might be injured and inserts an SPD) will be noted in the case report form and will not be excluded from the study. If the surgeon decides intraoperatively to perform a craniotomy (eg, due to clotted hemorrhage which does not evacuate with burr-hole trepanation), the patient will be excluded from the trial.

### Data Collection

To preserve confidentiality all patients are allocated a unique study identifier during the recruitment process that is used on all data collection forms. All study documentation is held in secure offices, and the study researchers operate according to a signed code of confidentiality. All data are entered into a password-secured database by the data managers.

### Participant Timeline

1. Patient admission.
2. Clinical evaluation and cranial CT (if none exists).
3. Obtain informed consent.
4. If the patient is treated with vitamin K antagonist, preoperative reversal using Beriplex and Konakion is done aiming for a preoperative international normalized ratio of 1.3. In patients treated with Aspirin Cardio or Plavix, the medications should be discontinued and surgery postponed for 5 to 7 days if possible. If emergency surgery is indicated, the Aspirin Cardio or Plavix should be discontinued preoperatively and for 2 to 6 weeks postoperatively depending on the indication for treatment (see step 12).
5. Surgical evacuation of the cSDH with burr-hole trepanation.
6. Randomization: SDD group versus SPD group.
7. Monitoring in the intermediate or intensive care unit.
8. Cranial CT and clinical evaluation 24 hours postoperative.
9. Low-weight molecular heparin in prophylactic dosage is given postoperatively with mobilization of the patient (with the drainage pinched off) after 24 to 48 hours.
10. Drain removal after 36 to 48 hours.
11. Patient discharge after clinical evaluation on postoperative day 5 or later.
12. Resumption of Aspirin Cardio or Plavix no earlier than 2 weeks postoperatively for patients with secondary prophylaxis and 6 weeks postoperatively for patients with primary prophylaxis. Resumption of Marcoumar should be no earlier than 6 weeks postoperatively.
13. Clinical evaluation and cranial CT at the outpatient clinic 6 weeks postoperatively (±2 weeks).
14. Clinical evaluation at the outpatient clinic 12 months postoperatively (±4 weeks).

**Figure 1:**
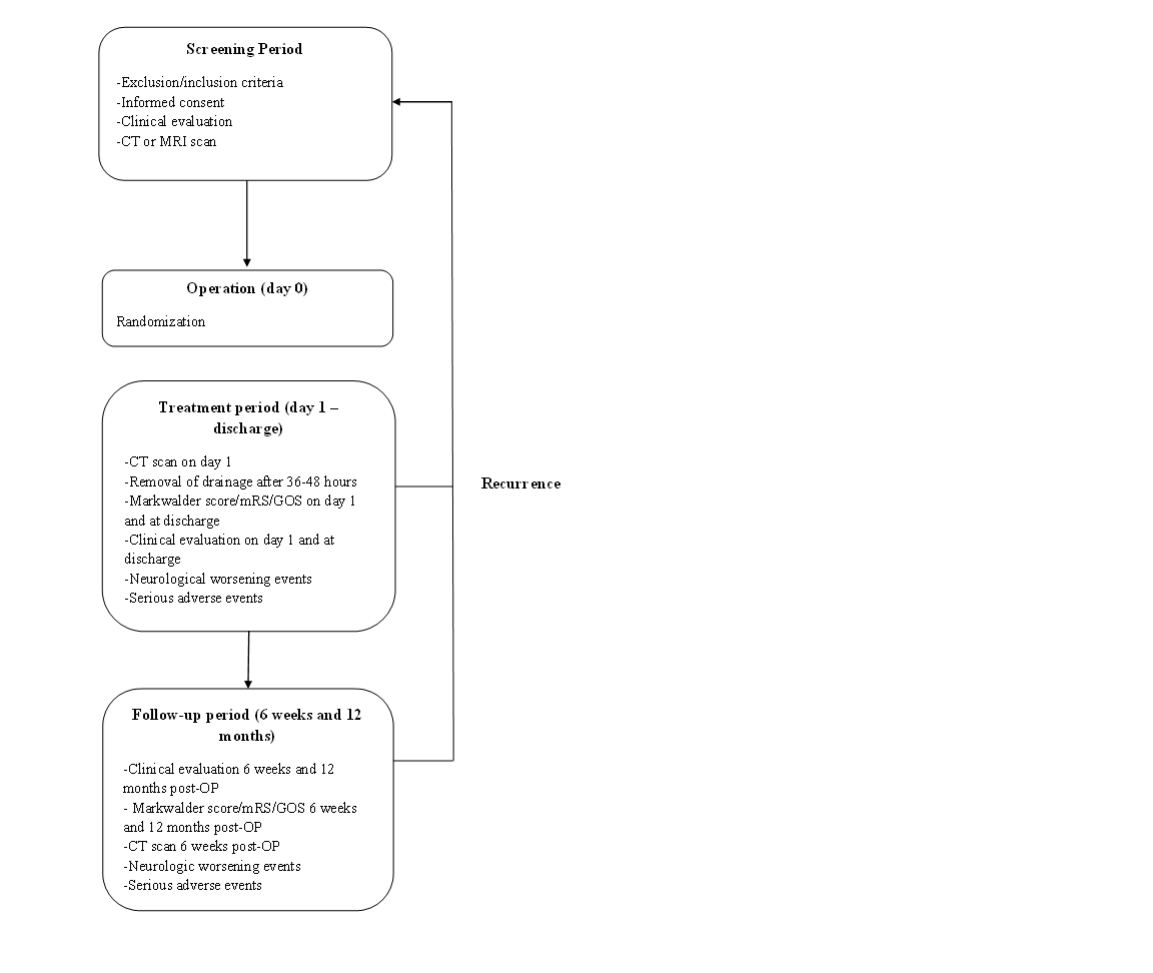
Study workflow mRS: modified Rankin Scale, GOS: Glasgow Outcome Scale, CT: computer tomography, MRI: magnetic resonance imaging.

See Multimedia Appendix 1 for a time schedule of enrollment, interventions, assessments, and visits for participants. The flow diagram (Figure 1) illustrates the key steps of the trial.

### Serious Adverse Events

Serious adverse events (SAEs) are recorded on the SAE form and include any of the following outcomes: death, life threatening events, requirement for a new hospitalization or prolongation of existing hospitalization, recurrent event, or persistent or significant disability caused by the surgical treatment. All SAEs will be reported to the local ethics committee, Ethikkommission Nordwest- und Zentralschweiz (EKNZ), within 7 days.

### Outcomes

The primary outcome measure is recurrence needing revision surgery within 12 months postoperatively. Secondary outcome measures include: (1) complication (morbidity) within 12 months postoperatively; (2) mortality within 12 months postoperatively; (3) Markwalder Scale, mRS, and Glasgow Outcome Score; and (4) radiological characteristics of postoperative CT images at 24 hours and 6 weeks. On axial CT scans, the midline shift is measured in millimeters at the level of the foramina of Monro and the thickness of the hematoma at the thickest area.

### Sample Size

Initially the study was planned as a superiority study to show a significant difference in the recurrence rate of cSDH between insertion of an SPD and an SDD. When estimating the sample size, a difference in the recurrence rate of 10% in the SDD group versus 20% in the SDP group was assumed (based on Bellut et al and Santarius et al [2,5]), leading to the estimate of 150 patients in each group. New studies [12] and the blind data review of the first 56 patients (both groups pooled) suggested a lower recurrence rate of 7%. Therefore the sample size was reviewed. At the same time, the study design was changed from a superiority to a noninferiority design, and a noninferiority margin of 3.5% was defined.

We reestimated the recurrence rates in a blinded manner based on the overall recurrence rate. Since no hypothesis test was performed, no *P* value adjustment to control type I error was needed. Data from all patients who had a follow-up visit could be used for the sample size review. When reestimating the recurrence rates, it was assumed that the probability of being in one group or the other (SDD vs SPD) for patients who had a follow-up visit would be equal. As stated the reestimated recurrence rate was 7%, which was the expected difference between the SDD group and SPD group (3.5%−10.5%=7%). Using the reestimated recurrence rates, the sample size N was reestimated using a resampling procedure. Each sample size, N_i=1;...;101_=50;...;150, was evaluated by sampling 9999 times N_i_ individual samples based on the assumptions described above. Confidence intervals (CIs) for the difference between proportions were calculated using a continuity-corrected modification of the Wilson score method. Sample size was set to ensure with 80% power (1–β=0.8) (ie, in 80 of 100 hypothetical repetitions of the study) the estimation of a 95% CI, which is entirely below the predefined noninferiority margin of 3.5%.

**Figure 2:**
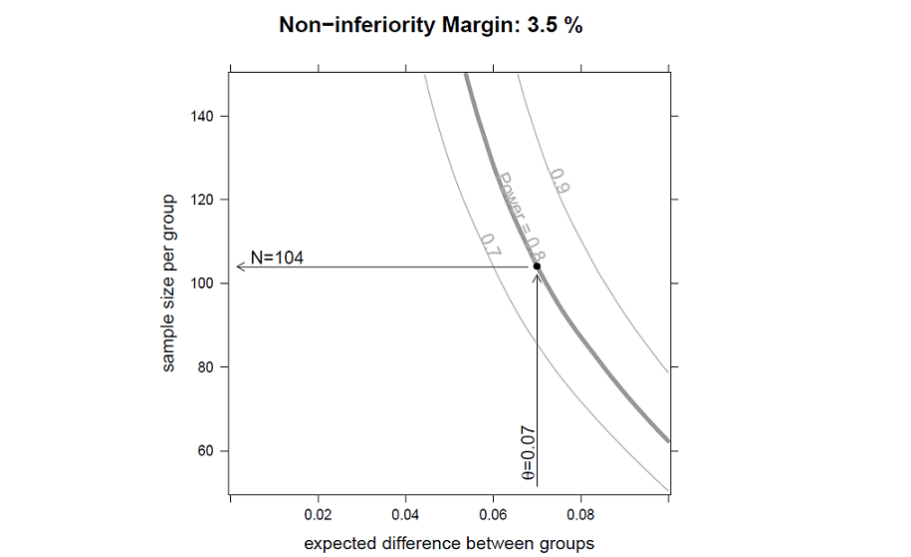
Sample size calculations.

For this study, a total of 220 patients should be randomized to ensure 208 evaluable patients (110 patients randomized per study arm) (Figure 2) considering an overall drop-out rate of 5% after randomization (eg, death, lost to follow-up).

### Statistical Analysis

Analysis will be done on an intention-to-treat basis. Given the possibility of a proportion of crossovers, a secondary sensitivity per-protocol analysis will be undertaken. The statistical analysis for the primary outcome measure will be done in a noninferiority design with 95% CI and a noninferiority margin of 3.5% between the groups, while the secondary measures will be analyzed in a superiority design, where a *P* value of less than .05 is considered statistically significant.

Patient data will be prospectively collected and registered on case report forms. Age; sex; date of trauma; blood thinners; medical history; GCS and neurological condition at admission; hematoma size; side; and the existence of brain herniation, hydrocephalus, and midline shift are documented as basic characteristics. Clinical outcome variables (24 hours postoperative, at discharge, 6 weeks postoperative, and 12 months postoperative): Glasgow Coma Scale and (improvement of) neurological condition, mRS, Glasgow Outcome Score, Markwalder score, recurrence needing reoperation, complications (eg, infection, epilepsy, aphasia, paresis), hospitalization time, and mortality. Radiological outcome variables (24 hours postoperative and 6 weeks postoperative): hematoma size, midline shift, and rebleed seen on cranial CT. Intraoperative variables: elective or emergency procedure, type of hematoma (chronic, acute, subacute), drain type, crossover, number of membranes, and existing communication between the two burr-holes.

### Monitoring

The trial master folder and case report form data for each participant will be inspected by a monitor at yearly intervals throughout the study to verify the completeness, consistency, and accuracy of the data. The existence and integrity of the informed consent forms signed by the patient or legal representative will be monitored as well. The study monitoring is provided by Kammermann Monitoring Service, Zug, Switzerland.

A strict confidential yearly interim analysis is done by a statistician (Clinical Trial Unit, Basel, Switzerland) for the recurrence rate (primary outcome measure) and morbidity (secondary outcome measure). The trial will be stopped if the intervention arm (SPD) shows a significant noninferiority margin of 3.5% compared to the treatment arm (SDD) or if recruitment rates are unexpectedly low.

### Ethical Issues

This study was approved by the local ethics committee (EKNZ, Basel, Switzerland, AG2013/001). The trial is conducted within the International Conference of Harmonization of Technical Requirements for Registration of Pharmaceuticals for Human Use Good Clinical Practice guidelines and the principles of the Declaration of Helsinki and is registered in the clinical study database ClinicalTrials.gov (NCT01869855).

## Results

The study is a currently ongoing study in two neurosurgical centers: Kantonsspital Aarau and University Hospital of Basel. Enrollment began in April 2013. We expect all study-related activities to be completed by the end of 2016 or beginning of 2017.

## Discussion

To date, evidence-based recommendations concerning the operative and postoperative treatment of cSDH are sparse. Most recommendations are based on observational or retrospective studies and some meta-analyses leading to class II or III recommendations [3]. For the surgical management of cSDH only one randomized controlled trial (RCT) exists, providing grade I evidence and showing that the intraoperative insertion of a drain after the completion of a burr-hole trepanation reduces the recurrence rates of cSDH significantly [2]. Further RCTs investigating and scrutinizing the standard treatment of cSDH are warranted, first and foremost due to the fact of a steady increase in the incidence of cSDH as a result of prolonged life expectancy [2,3]. With this multicenter RCT, we intend to provide grade I evidence (and class I recommendations) to an additional aspect in the surgical treatment of cSDH, namely the ideal and safest localization of an intraoperative drain. In our opinion this RCT will have great impact on the surgical management of patients presenting with this frequent condition. We hope that this clinical study will contribute to the important goal of evidence-based treatment of cSDH.

## Multimedia Appendix 1

Visit assessment schedule.

## Abbreviations

CI: confidence interval
CT: computed tomography
cSDH: chronic subdural hematoma
MRI: magnetic resonance imaging
mRS: modified Rankin Scale
RCT: randomized clinical trial
SAE: serious adverse events
SDD: subdural drain
SPD: subperiosteal drain
