# Supplementary material for: Use of Subperiosteal Drain Versus Subdural Drain in Chronic Subdural Hematomas Treated With Burr-Hole Trepanation: Study Protocol for a Randomized Controlled Trial
Source: JMIR Res Protoc. 2016 Apr 8;5(2):e38. doi: 10.2196/resprot.5339 (PMC4841895; doi:10.2196/resprot.5339)
Supplement: Supplementary file 1 [file resprot_v5i2e38_app1.pdf]

Table 1 Visit assessment schedule

Visits	Screening	During the procedure	Directly after the procedure	1st post-OP day	Discharge	6 weeks post OP (± 2 weeks)	1 year post OP (± 4 weeks)
Informed consent	x						
Medical history/demographics	x						
Blood thinners	x		x		x	x	x
Physical examination	x			x	x	x	x
Markwalder score/mRS/GOS	x			x	x	x	x
LOS					x		
Randomization		x					
Surgical procedure[1]			x				
Recurrence				x	x	x	x
Neurologic worsening events				x	x	x	x
CT scan/MRI scan	x[2]			x[3]		x[3]	

Serious adverse events	x	x	x	x	x

mRS: modified Rankin Scale
GOS: Glasgow Outcome Scale
LOS: length of stay
CT: computer tomography
MRI: magnetic resonance imaging
Post-OP: postoperative

[1] procedure date, type of inserted drainage (subdural or subperiosteal), cross-over patients, blood thinners during or before the procedure and if they were reverted medically of through time, presence of membranes in the hematoma cavity, communication between both burr-holes will be recorded in the CRF

[2] mild line shift measured at the level of the foramina of Monro, maximal hematoma width, hematoma side will be recorded in the CRF

[3] mild line shift measured at the level of the foramina of Monro, maximal hematoma width, new intracranial bleeding will be recorded in the CRF. In case of neurological worsening CT scans will be repeated as required, yet these results will be not recorded in the CRF.
